# Exploration of Students’ Perception of Academic Misconduct: Do Individual Factors, Moral Philosophy, Behavioral Intention, and Judgment Matter?

**DOI:** 10.3389/fpsyg.2022.857943

**Published:** 2022-04-05

**Authors:** Chiao Ling Huang, Shu-Ching Yang, Chun-An Yang

**Affiliations:** ^1^Faculty of Education, Department of Educational Information Technology, East China Normal University, Shanghai, China; ^2^Institute of Education, The Intelligent Electronic Commerce Research Center, National Sun Yat-sen University, Kaohsiung, Taiwan

**Keywords:** academic dishonesty, moral philosophy, ethical judgment, behavioral intention, higher education

## Abstract

Using Reidenbach and Robin’s Multidimensional Ethics Scale, this study designs three scenarios related to academic dishonesty (AD) dilemmas to explore students’ moral philosophies, behavioral intentions, and ethical judgments and further examines whether students with different individual factors (i.e., culture of place, gender, and educational level) have differences in the above variables. A total of 605 students from two areas, Taiwan and Mainland China, participated in this study. The results indicated that Taiwanese students had stricter moral equity, relativism, and contractualism philosophies in the duplicate submission scenario than Mainland China students. They also had stricter moral equity and relativism philosophies in the incomplete citation scenario. Similarly, relatively harsh relativism and contractualism philosophies accompanied by a low level of willingness to be a perpetrator in the failure to cite research published in other countries scenario were found. In addition, females applied relatively harsh moral equity and utilitarianism to all scenarios, reporting that they and their peers were less likely to engage in all AD activities. Graduates had a stricter egoism attitude toward duplicate submission and had stricter moral equity, relativism, and contractualism philosophies toward the behavior of incomplete citation. Graduate students also had strict moral equity, relativism, egoism, and contractualism beliefs in the failure to cite the foreign research scenario. Finally, regression analysis showed that moral equity, contractualism, and self-behavioral intention are significant predictors of students’ ethical evaluations in the three scenarios.

## Introduction

Muñoz-García and Aviles-Herrera (2014) defined academic dishonesty (AD) as students’ behaviors that are not aligned with reason, ethical standards, or the values that are considered good in a given culture. Such behavior continues to increase and grow rampant in the learning environment, becoming a concern in higher education (Şendağ et al., 2012; Taradi et al., 2012; Simola, 2017; Krou et al., 2021).

From the perspective of decision-making, individual behavior is a decision that is intertwined with multiple factors and the rationale to engage in AD may be complicated beyond our expectations. In particular, researchers have found that students use more than one rationale or philosophy when making ethical judgments, and the significance of these factors may differ across ethical situations (Cohen and Pant, 1998; Jung, 2009; Yang, 2012; Bratton and Strittmatter, 2013). Nevertheless, few studies discuss this issue in the AD field, forming an academic gap that impedes us from fully recognizing students’ academic misconduct.

Fortunately, the application of the Multidimensional Ethics Scale (MES) can help us address this inadequacy. The MES, developed by Reidenbach and Robin (1988), provides insights into the philosophies that underlie the ethical decisions made by students who engage in AD and has been successfully applied to previous AD studies (i.e., Yang, 2012; Strittmatter and Bratton, 2014). Reidenbach and Robin (1988) established a foundation for ethical evaluation by identifying five major dimensions of the “ethical–unethical” construct. These dimensions can be used to evaluate an individual’s ethical judgments with respect to AD. The five philosophies of MES are moral equity, relativism, egoism, utilitarianism, and contractualism.

Moral equity refers to an individual’s perception of fairness, rightness, and justice. Relativists argue that ethical rules are based on guidelines and parameters embedded in the sociocultural system rather than in the individual; therefore, ethical standards are different across cultures and societies and are shaped by the individual’s prevailing culture and normative behaviors. In contrast to egoism, which compels individuals to act for self-interest or personal benefits (i.e., a desire for success or a promotion that is stronger than the ethical constraints being violated), utilitarianism stresses the extent to which an action leads to the greatest good for society. Contractualism refers to the perception of justice based on individuals’ conceptions of their duties (i.e., their perceptions of justice), implied contracts, or unwritten obligations (i.e., corporate codes of ethics and academic honor codes).

The MES has long been used to study ethical issues (e.g., Gupta, 2010; Williamson et al., 2011; Bratton and Strittmatter, 2013; Leonard and Jones, 2017; Iqbal, 2019). It usually employs a scenario-type survey to assess respondents’ moral standards. Participants are presented with an ethical scenario ending with a specific action and are then asked to judge the action according to several ethical perspectives (Eyal et al., 2011). The hypothetical nature of the scenario method minimizes the sense of judgment and the social desirability effect (Fowler, 1989) and thus provides an advantage over self-reports of actual violations (Schuhmann et al., 2013). It has also been successfully applied to explore AD (i.e., Yang, 2012).

Notably, when inspecting the impact of moral values on behavior, behavioral intention and judgment of events should be considered together. Behavioral intention, as a central factor of action, represents the degree that is increased to perform a deed, and this is the mind’s direction in the behavioral decision-making process (Koenig et al., 2019). Judgment reflects the individual’s attitude on something, and the outcome usually depends on the individual’s moral foundations. Evidence has shown that intention or judgments influence an individual’s action even in deviant behaviors. For example, Martin et al. (2010) found that gambling intention and gambling frequency have a close-knit connection.

In addition, demographic characteristics (such as culture of place, gender, and educational level) as the basic attributes of an individual have gradually been discussed in AD research. For culture, studies have found that students with different cultures have different engagement frequencies and perceptions of unethical academic acts (Hamlen, 2012). Given, the culture plays an influential role on both moral foundations and judgments (Cantarero et al., 2021), academic misconduct that is viewed as dishonest in one cultural context may be appropriate in another (Yukhymenko-Lescroart, 2014). In such a learning atmosphere and environment, students from Taiwan and Mainland China may have similar values with minor differences in learning, and this deserves exploration.

For gender, the majority of studies found that women had fewer AD behaviors (Sideridis et al., 2016; Akbasli et al., 2019; Peled et al., 2019), lower behavioral intentions to engage in AD activity (Koul, 2012; Peled et al., 2019) and less willingness to report others’ AD transgression (Simon et al., 2004) than men. Differences in age and education level were also found to influence students’ AD actions (e.g., Brown et al., 2020; Tremayne and Curtis, 2021). Apparently, examining the individual factors seems to be needed.

Nevertheless, although researchers noticed the possible factors mentioned earlier, they never considered them at the same time, resulting in the knowledge obtained being fragmentary, trivial, and incomplete. Since ethical principles and standards are critical elements in any professional field (Iqbal, 2019), MES can improve our understanding of the moral basis of individuals’ decisions. It is expected that applying MES to extend the exploration of academic ethics would be fruitful.

Therefore, this study designs three moral dilemmas to examine the differences in moral philosophies, behavioral intentions, and judgment in different demographic groups by using the MES. Specifically, we attempt to answer the following questions: (1) Does students’ moral philosophy vary depending on the type of AD? (2) Do students with different traits (i.e., culture of place, gender, and educational level) exhibit dissimilar AD actions? (3) Does moral philosophy influence students’ AD behavioral intentions and their judgments?

## Materials and Methods

### Research Framework

**Figure 1:**
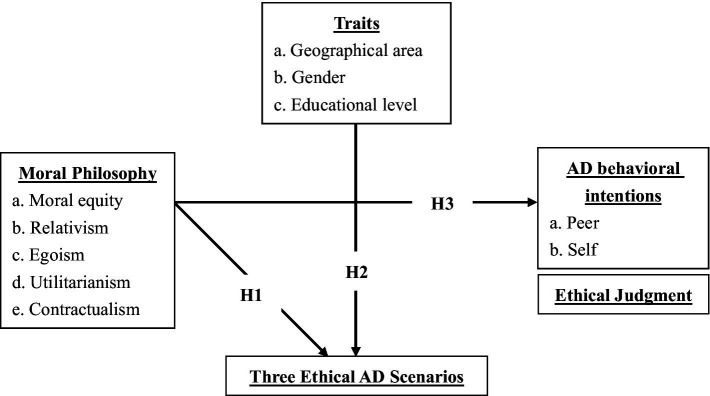
Research hypothesis framework.

This paper develops the following hypothesis form the research framework (see Figure 1).

*Hypothesis 1 (H1)*: Students’ moral philosophy differs among the three types of AD scenarios.

*Hypothesis 2 (H2)*: There will be differences between the personal traits (a: geographical area, b: gender, and c: educational level) and AD of the three ethical scenarios.

*Hypothesis 3 (H3)*: Moral philosophy will influence students’ AD behavioral intentions and judgments.

### Participants

#### Target Sample

We recruited Taiwanese students and Chinese students as the target sample in this study because students from these two areas have specific and subtle differences in cultural values. Taiwan and Mainland China are dominated by the Han ethnic group and are deeply imbued with Confucianism. Under the influence of Confucianism, people in both areas consider education to be important, and the percentage of people attending universities is relatively high. Nevertheless, Taiwanese have been heavily influenced by Western culture due to history and education reforms. This is also reflected in Taiwan’s higher education system, which emphasizes autonomy and academic freedom, and the learning culture is more concerned with what students learn than with obtaining a degree. In contrast, in Mainland China, schools establish a strict graduation year at the higher education level to urge students to graduate on time; if students delay graduation, s/he and the advisor will receive attention from the school level. This shows that the two areas have different orientations on higher education.

#### Procedure

We adopted a convenience sampling technique in this study, and all data were collected via a paper-and-pencil questionnaire on September 2021. We contacted familiar instructors to request them to help distribute the questionnaires. Before distributing the questionnaires, instructors clearly informed students of the research content, including purpose, procedure, and requirement. The participants involved in the research surveys were all voluntary, anonymous, and confidential; in addition, participants’ class grades would not be affected by whether they joined. Those who participated in this investigation were asked not to write down any personal information on the questionnaire and submitted the questionnaire by themselves. Additionally, they can drop out at any time without penalties. Finally, 605 social science majors completed the questionnaire, and only 6 participants could not finish the questionnaire (i.e., the dropout rate was approximately 1%). Of them, 53.6% are from Mainland China, and 46.4% are from Taiwan. Male and female students made up 48.3% and 51.7% of the sample, respectively, and the average age was 21.8 years.

### Instrument

We adopted the MES (Jung, 2009), which uses scenario-type item descriptions to assess students’ moral philosophy, behavioral intentions, and judgments.

#### Three Ethical Scenarios

Regarding the component of scenarios, since university students and graduate students are at different educational stages that focus on developing different competencies, to compare the differences between these two kinds of students, the questionnaire design selected the learning experiences that they both have. Considering that university programs in both Taiwan and Mainland China offer mandatory courses on how to conduct research, in particular, all universities in Mainland China and some universities in Taiwan require students to complete a thesis before graduation. Therefore, we designed scenarios that can reflect realistic ethical dilemmas faced by students based on their learning experience, and three scenarios related to academic writing were developed.

Each scenario was presented in Mandarin Chinese and consisted of an ethical dilemma and an action taken by a hypothetical student (see Table 1). *Scenario A—Duplicate submission* presents an example of resubmitting the same work to different publishers without permission. *Scenario B—Incomplete citation* highlights the issue of plagiarism by describing a deliberate failure to appropriately cite one’s sources. *Scenario C—Failure to cite research published in other countries* presents another example of plagiarism by depicting a direct translation and revision of others’ work without attributing it to the authors.

**Table 1:** Descriptions of the three AD scenarios.

**Scenario A: Duplicate submission**
Jim submitted a paper to a journal for consideration for publication. Later, Jim decided to resubmit the paper to other journals, as the paper has been under review for a year and he had not received a response from the journal editor. Therefore, Jim resubmitted the original paper to other journals with hopes of improving its chances of being accepted and to see which journal would accept it first **Action:** Jim thinks it is ok to submit his paper to other journals while it is under review because he has not heard from the first journal editor despite waiting for a long time
**Scenario B: Incomplete citation**
Ken prepared to submit a proposal to a conference. He collected many useful materials from websites and journal articles for the paper. However, he found that he forgot to give citations when he finished up the paper. Unfortunately, he did not have much time to find the sources, as the deadline was just around the corner **Action:** Ken thinks that he will not be able to complete this paper if he excludes the references for the findings and content of some of these materials. He decides to use these materials despite failing to cite the reference sources properly. Other journal articles from which he cited are listed in the references
**Scenario C: Failure to cite research published in other countries**
May designed a questionnaire for her study by consulting research reports and questionnaires from domestic and international databases. She believed that she only needed to cite the domestic reference sources, as the questionnaires that she consulted from other countries had been translated and revised and were different from the originals, so there was no need to refer to those international reference sources in her paper **Action:** In her study, May only lists the domestic reference sources and omits the international reference sources

Furthermore, to ensure that the content of the AD scenario is not biased, a process of conceptual clarification and phrasing (i.e., the scenarios used vocabulary and wording that minimized any sense of judgment) is applied to minimize the social desirability effect. Two specialist professors, one research fellow, two graduates, and college students were invited to test the content validity of these three scenarios. All of this also helped us ensure that descriptions, questions, and response options were meaningful and avoided biased responses from participants.

#### Multidimensional Ethics Scale

The MES used to measure AD in this research is based on Jung’s (2009) study. The scale uses a 7-point Likert format and comprises 15 items: four items measuring moral equity, two measuring relativism, two measuring egoism, two measuring utilitarianism, and two measuring contractualism (1 = consistent with the philosophy to 7 = not consistent with the philosophy). In addition to the 12 items assessing the five philosophies, three questions are used to evaluate students’ overall ethical judgment of the actions taken (1 = ethical to 7 = unethical) and to determine the behavioral intentions of the student (self) and his or her peers (1 = would perform the action to 7 = would not perform the action).

Cronbach’s alpha values for the three scenarios were 0.92, 0.91, and 0.93 for moral equity; 0.85, 0.92, and 0.92 for relativism; 0.72, 0.70, and 0.75 for egoism; 0.78, 0.71 and 0.74 for utilitarianism; 0.82, 0.91, and 0.91 for contractualism; and 0.90, 0.93, and 0.95 for Scenarios A, B and C, respectively. All of the internal consistency reliability estimates were well above the recommended cutoff of 0.70 (Nunnally, 1978; Rovai et al., 2014; Heale and Twycross, 2015).

### Statistical Analyses

Four statistical techniques were adopted to analyze the collected data. To address the first research question and hypothesis, we conducted repeated-measures ANOVA to assess whether the specific moral standard of students varied across the scenarios. Also, to further compare the differences between students’ perceived behavioral intentions and those of their peers, the paired t-test was used in this study. To address the second research question and hypothesis, we used an independent t-test to compare the intensity of specific moral standards possessed by students with different demographic characteristics in each scenario. To address the third research question and hypothesis, multiple regression analysis was used to scrutinize the predictive power of variables on behavioral intentions and judgment in each scenario.

## Results

### Ethical Philosophy, Judgments, and Behavioral Intentions in the Three Scenarios

The results indicated that the intensity of moral concepts varied across the scenarios (Moral equity: *F* = 536.26, *p* < 0.001, $\eta_{p}^{2}$ = 0.47; Relativism: *F* = 313.15, *p* < 0.001, $\eta_{p}^{2}$ = 0.34; Egoism: *F* = 234.95, *p* < 0.001, $\eta_{p}^{2}$ = 0.28; Utilitarianism: *F* = 210.32, *p* < 0.001, $\eta_{p}^{2}$ = 0.26; Contractualism: *F* = 201.27, *p* < 0.001, $\eta_{p}^{2}$ = 0.25). With this, Hypothesis 1 was supported. Specifically, students had stricter beliefs in all the philosophies in scenario B and C than in scenario A.

Students also exhibited latitude toward the action in scenario A. They believed that both themselves (*F* = 217.96, *p* < 0.001, $\eta_{p}^{2}$ = 0.27; *M*_a_ = 3.73, *M*_b_ = 5.15, *M*_c_ = 5.24) and their peers (*F* = 233.16, *p* < 0.001, $\eta_{p}^{2}$ = 0.27; *M*_a_ = 3.28, *M*_b_ = 4.53, *M*_c_ = 4.74) were more likely to engage in duplicate submission, rating this act as relatively ethical (*F* = 233.83, *p* < 0.001, $\eta_{p}^{2}$ = 0.27; *M*_a_ = 4.53, *M*_b_ = 5.93, *M*_c_ = 5.75).

To thoroughly understand students’ intention and their perceptions of their peers, we also conducted a paired t-test and found that students perceived that they were more honest (*M*_a_ = 3.73, *M*_b_ = 5.14, *M*_c_ = 5.24) than their counterparts (*M*_a_ = 3.29, *M*_b_ = 4.53, *M*_c_ = 4.74) in all scenarios (*t*_a_ = 8.69, *p* < 0.001; *t*_b_ = 11.45, *p* < 0.001; *t*_c_ = 10.25, *p* < 0.001).

### Geographical Area, Gender, and Education Level Differences in the Three Scenarios

#### Geographical Area

Table 2 shows that students from the two geographical areas differed in moral equity (*t* = −2.41, *p* = 0.016, *d* = −0.20), relativism (*t* = −5.93, *p* < 0.001, *d* = −0.49), and contractualism (*t* = −2.52, *p* = 0.012, *d* = −0.21) in scenario A. These findings suggest that Taiwanese students’ judgment of duplicate submissions was affected by fairness, the external guidelines embedded in the social/cultural system and individual attitudes toward obligations.

**Table 2:** Geographical area differences in responses across AD scenarios.

Scenario	A				B				C			
	Duplicate submission				Incomplete citation				Failure to cite research published in other countries			
MES	Area	*M*	*SD*	*t*	*d*	*M*	*SD*	*t*	*d*	*M*	*SD*	*t*	*d*
Moral equity^a^	China	3.53	1.40	−2.41^*^	−0.20	5.17	1.29	−3.45^**^	−0.28	5.32	1.18	−1.46	−0.12
	Taiwan	3.81	1.38			5.50	1.03			5.46	1.14		
Relativism^a^	China	3.51	1.43	−5.93^***^	−0.49	5.17	1.46	−3.79^***^	−0.31	5.02	1.42	−3.41^**^	−0.28
	Taiwan	4.22	1.46			5.58	1.17			5.40	1.26		
Egoism^a^	China	3.33	1.40	0.53	0.04	4.48	1.32	−0.24	−0.02	4.39	1.45	−0.29	−0.02
	Taiwan	3.27	1.39			4.51	1.44			4.42	1.46		
Utilitarianism^a^	China	2.94	1.55	1.02	0.08	3.91	1.63	−1.61	−0.13	4.07	1.57	−1.07	−0.09
	Taiwan	2.81	1.53			4.13	1.74			4.21	1.67		
Contractualism^a^	China	4.47	1.66	−2.52^*^	−0.21	5.74	1.40	−1.85	−0.15	5.58	1.36	−2.54^*^	−0.21
	Taiwan	4.81	1.56			5.94	1.19			5.84	1.15		
Behavioral intentions (Self)^b^	China	3.68	1.80	−0.81	−0.06	5.06	1.67	−1.56	−0.13	5.08	1.63	−3.05^**^	−0.26
	Taiwan	3.80	1.92			5.27	1.60			5.49	1.53		
Behavioral intentions (Peer)^b^	China	3.21	1.54	−1.44	−0.12	4.49	1.66	−0.89	−0.07	4.71	1.56	−0.67	−0.05
	Taiwan	3.40	1.56			4.61	1.62			4.79	1.60		
Ethical judgment^c^	China	4.48	1.82	−1.03	−0.08	5.90	1.28	−0.90	−0.07	5.72	1.39	−0.64	−0.06
	Taiwan	4.63	1.74			5.99	1.27			5.80	1.33		

a 7 = not consistent with the philosophy to 1 = consistent with the philosophy.

b 7 = would not perform the action to 1 = would perform the action.

c 7 = unethical to 1 = ethical; d represents Cohen’s d.

* *p* < 0.05;

** *p* < 0.01;

*** *p* < 0.001.

For scenario B, students exhibited differences in moral equity (*t* = −3.45, *p* = 0.001, *d* = −0.28) and relativism (*t* = −5.79, *p* < 0.001, *d* = −0.31). Taiwanese students viewed plagiarism with a stricter sense of moral equity and relativism, basing their evaluation of this act more on a comprehensive consideration of individual and social culture. In other words, in addition to the subjective perception of plagiarism as a violation of social justice, strictures against such behavior in the given society and culture were also considered.

For scenario C, students displayed dissimilar attitudes in terms of relativism (*t* = −3.41, *p* = 0.001, *d* = −0.28) and contractualism (*t* = −2.54, *p* = 0.011, *d* = −0.21), revealing that Taiwanese students thought that an individual’s behavior should meet cultural expectations and that individuals should abide by conventional rules, even in the absence of regulation by the law or government. Notably, this is the only scenario in which Taiwanese students saw themselves as less likely to engage in such activities than Mainland Chinese students (*t* = −3.05, *p* = 0.002, *d* = −0.26). Given the above, Hypothesis 2a was mostly supported.

#### Gender

It was found that females viewed the duplicate submissions scenario more negatively than males, and these perceptions were based on the philosophies of moral equity (*t* = −2.59, *p* = 0.010, *d* = −0.23), utilitarianism (*t* = −2.97, *p* = 0.003, *d* = −0.27), and contractualism (*t* = −2.56, *p* = 0.011, *d* = −0.23). Women considered this form of AD to be inconsistent with social justice, as not bringing the greatest benefits to society and as conflicting with individual criteria and obligations. Women also saw themselves (*t* = −3.00, *p* = 0.003, *d* = −0.27) and their peers (*t* = −2.00, *p* = 0.046, *d* = −0.18) as less likely to engage in this type of misconduct and strongly stated that it is unethical (*t* = −3.55, *p* < 0.001, *d* = −0.32).

For scenario B, females were more likely to see plagiarism as a violation of moral equity (*t* = −4.44, *p* < 0.001, *d* = −0.40), relativism (*t* = −2.00, *p* = 0.047, *d* = −0.17), egoism (*t* = −3.17, *p* = 0.002, *d* = −0.29), and utilitarianism (*t* = −2.32, *p* = 0.021, *d* = −0.20). In other words, compared to males, females felt that incomplete citation was more unjust, socially or traditionally unacceptable, and unbalanced between the personal or social costs and the results obtained. They rated this act as a violation of academic ethics (*t* = −3.08, *p* = 0.002, *d* = −0.29) and reported a low possibility of their own (*t* = −2.47, *p* = 0.014, *d* = −0.22) and peer (*t* = −2.54, *p* = 0.011, *d* = −0.23) engagement in such an activity.

For scenario C, gender differences existed in moral equity (*t* = −2.73, *p* = 0.007, *d* = −0.25), egoism (*t* = −2.95, *p* = 0.003, *d* = −0.27), utilitarianism (*t* = −2.59, *p* = 0.010, *d* = −0.23), contractualism, (*t* = −2.61, *p* = 0.009, *d* = −0.23), self-intention (*t* = −2.95, *p* = 0.003, *d* = −0.24), peer intention (*t* = −2.16, *p* = 0.031, *d* = −0.20), and ethical judgment (*t* = −3.31, *p* = 0.001, *d* = −0.31).

For females, the failure to cite research published in other countries was relatively unethical and was obviously morally wrong, not only because it was in violation of the norms and responsibilities of individuals or organizations but also because it does not produce gains for individuals or society. Both they and their peers were perceived to be less likely to engage in this kind of misconduct, and this act was also fundamentally unethical. Given the above, Hypothesis 2b was mostly supported (Table 3).

**Table 3:** Gender differences in responses across AD scenarios.

Scenario	A				B				C			
	Duplicate submission				Incomplete citation				Failure to cite research published in other countries			
MES	Gender	*M*	*SD*	*t*	*d*	*M*	*SD*	*t*	*d*	*M*	*SD*	*t*	*d*
Moral equity^a^	M	3.41	1.40	−2.59^**^	−0.23	4.97	1.19	−4.44^***^	−0.40	5.18	1.10	−2.73^**^	−0.25
	F	3.73	1.38			5.44	1.18			5.46	1.18		
Relativism^a^	M	3.70	1.60	−1.03	−0.09	5.16	1.25	−2.00^*^	−0.17	5.09	1.36	−0.85	−0.08
	F	3.84	1.44			5.39	1.42			5.20	1.39		
Egoism^a^	M	3.18	1.38	−1.38	−0.13	4.21	1.37	−3.17^**^	−0.29	4.12	1.45	−2.95^**^	−0.27
	F	3.36	1.40			4.60	1.36			4.51	1.44		
Utilitarianism^a^	M	2.60	1.55	−2.97^**^	−0.27	3.74	1.79	−2.32^*^	−0.20	3.84	1.67	−2.59^*^	−0.23
	F	3.01	1.52			4.09	1.63			4.21	1.57		
Contractualism^a^	M	4.35	1.64	−2.56^*^	−0.23	5.66	1.39	−1.82	−0.16	5.47	1.31	−2.61^**^	−0.23
	F	4.72	1.60			5.88	1.30			5.77	1.27		
Behavioral intentions (Self)^b^	M	3.38	1.80	−3.00^**^	−0.27	4.88	1.62	−2.47^*^	−0.22	4.96	1.64	−2.95^**^	−0.24
	F	3.88	1.85			5.24	1.64			5.35	1.57		
Behavioral intentions(Peer)^b^	M	3.08	1.55	−2.00^*^	−0.18	4.26	1.64	−2.54^*^	−0.23	4.51	1.58	−2.16^*^	−0.20
	F	3.36	1.53			4.64	1.64			4.82	1.56		
Ethical judgment^c^	M	4.15	1.88	−3.55^***^	−0.32	5.66	1.42	−3.08^**^	−0.29	5.44	1.54	−3.31^**^	−0.31
	F	4.72	1.72			6.04	1.21			5.88	1.27		

a 7 = not consistent with the philosophy to 1 = consistent with the philosophy.

b 7 = would not perform the action to 1 = would perform the action.

c 7 = unethical to 1 = ethical; d represents Cohen’s d.

* *p* < 0.05;

** *p* < 0.01;

*** *p* < 0.001.

In summary, in these three scenarios, women generally had more stringent ethical standards with respect to AD (women have 3–4 stronger moral philosophies intensity than men in each scenario) and lower behavioral intentions (for both themselves and their peers) than men. The findings regarding gender differences in ethical judgment are consistent with those of Cohen and Pant (1998).

#### Education Level

Students’ perceptions of AD were affected by their education level. Students’ moral philosophy, their intention, and ethical judgment scores in scenario A were much lower compared with other scenarios, implying that students have relatively high tolerance and lenient standards for duplicate submission. Notably, graduates had a harsher egoism standard toward duplicate submission than undergraduates here (*t* = −2.47, *p* = 0.014, *d* = −0.22).

In scenario B, graduate students exhibited stricter moral equity (*t* = −3.16, *p* = 0.002, *d* = −0.29), relativism (*t* = −3.04, *p* = 0.002, *d* = −0.29), and contractualism (*t* = −1.99, *p* = 0.047, *d* = −0.19) beliefs than undergraduates. In essence, graduate students perceived plagiarism as wrong and inconsistent with social norms and duty. In addition, they also considered themselves (*t* = −2.43, *p* = 0.015, *d* = −0.19) to be less likely to engage in this act and judged plagiarism to be more unethical than undergraduates did (*t* = −2.94, *p* = 0.004, *d* = −0.28).

Finally, graduate students tended to apply the stricter philosophies of moral equity (*t* = −3.61, *p* < 0.001, *d* = −0.34), relativism (*t* = −3.32, *p* = 0.001, *d* = −0.32), egoism (*t* = −3.01, *p* = 0.003, *d* = −0.28), and contractualism (*t* = −2.49, *p* = 0.013, *d* = −0.23) to the behavior in scenario C (failure to cite research published in other countries). They also considered themselves (*t* = −1.98, *p* = 0.049, *d* = −0.18) to be less likely to engage in this act and tended to rate it as a form of AD (*t* = −3.37, *p* = 0.001, *d* = −0.32). Regardless of the philosophy (except utilitarianism) used to judge this action, it appeared to them to do more harm than good. Given the above, Hypothesis 2c was mostly supported (Table 4).

**Table 4:** Educational level differences in responses across AD scenarios.

Scenario	A				B				C			
	Duplicate submission				Incomplete citation				Failure to cite research published in other countries			
MES	Status	*M*	*SD*	*t*	*d*	*M*	*SD*	*t*	*d*	*M*	*SD*	*t*	*d*
Moral equity^a^	Under	3.60	1.32	−0.73	−0.07	5.21	1.20	−3.16^**^	−0.29	5.28	1.18	−3.61^***^	−0.34
	Grad	3.70	1.57			5.56	1.21			5.66	1.05		
Relativism^a^	Under	3.79	1.39	−0.08	−0.01	5.22	1.37	−3.04^**^	−0.29	5.05	1.37	−3.32^**^	−0.32
	Grad	3.80	1.72			5.61	1.36			5.48	1.34		
Egoism^a^	Under	3.21	1.34	−2.47^*^	−0.22	4.43	1.30	−1.48	−0.14	4.28	1.41	−3.01^**^	−0.28
	Grad	3.53	1.51			4.63	1.55			4.69	1.52		
Utilitarianism^a^	Under	2.90	1.46	1.03	0.10	3.96	1.58	−0.27	−0.03	4.05	1.54	−1.33	−0.12
	Grad	2.74	1.71			4.01	1.94			4.25	1.78		
Contractualism^a^	Under	4.61	1.54	0.27	0.03	5.75	1.30	−1.99^*^	−0.19	5.61	1.27	−2.49^*^	−0.23
	Grad	4.56	1.85			6.00	1.40			5.91	1.32		
Behavioral intentions (Self)^b^	Under	3.66	1.76	−1.13	−0.11	5.04	1.56	−2.43^*^	−0.19	5.16	1.57	−1.98^*^	−0.18
	Grad	3.88	2.09			5.41	1.75			5.45	1.66		
Behavioral intentions(Peer)^b^	Under	3.25	1.46	−0.55	−0.05	4.47	1.56	−1.20	−0.09	4.69	1.50	−1.08	−0.10
	Grad	3.33	1.76			4.67	1.86			4.86	1.78		
Ethical judgment^c^	Under	4.47	1.73	−1.50	−0.14	5.84	1.31	−2.94^**^	−0.28	5.64	1.37	−3.37^**^	−0.32
	Grad	4.73	1.93			6.18	1.20			6.07	1.30		

a 7 = not consistent with the philosophy to 1 = consistent with the philosophy.

b 7 = would not perform the action to 1 = would perform the action.

c 7 = unethical to 1 = ethical; d represents Cohen’s d.

* *p* < 0.05;

** *p* < 0.01;

*** *p* < 0.001.

### The Predictive Power of Moral Philosophy for Behavioral Intentions and Ethical Dimensions

Multiple regression analyses demonstrated that the moral philosophy variables predicted students’ judgments of their own and their peers’ behavioral intentions as well as their own ethical judgments across the scenarios (*F* = 54.63 ∼ 136.42, *ps* < 0.001) with an overall explanatory power ranging from 31 to 61%. These results showed that these predictor variables had moderate predictive power in the three scenarios, which Hypothesis 3.

An examination of all the peer behavioral intention models revealed that moral equity (*β*_A_ = 0.28, *β*_B_ = 0.16, *β*_C_ = 0.15), relativism (*β*_A_ = 0.17, *β*_B_ = 0.17, *β*_C_ = 0.25), and utilitarianism (*β*_A_ = 0.23, *β*_B_ = 0.19, *β*_C_ = 0.21) emerged as common indicators. Regarding self-behavioral intentions, moral equity (*β*_A_ = 0.26, *β*_B_ = 0.30, *β*_C_ = 0.20), relativism (*β*_A_ = 0.16, *β*_B_ = 0.14, *β*_C_ = 0.21), utilitarianism (*β*_A_ = 0.21, *β*_B_ = 0.22, *β*_C_ = 0.19), and contractualism (*β*_A_ = 0.21, *β*_B_ = 0.16, *β*_C_ = 0.24) emerged as common indicators, showing that these moral philosophies influence students’ intention to engage in these three types of AD.

For the models of ethical judgment, moral equity (*β*_A_ = 0.31, *β*_B_ = 0.27, *β*_C_ = 0.35), contractualism (*β*_A_ = 0.26, *β*_B_ = 0.38, *β*_C_ = 0.32), and self-behavioral intentions (*β*_A_ = 0.28, *β*_B_ = 0.25, *β*_C_ = 0.29) emerged as common indicators. Thus, the more unjust the behavior, the lower its compliance with social norms, and the less likely students themselves were to engage in it, the greater the students perceived that the act was not consistent with academic integrity (Table 5).

**Table 5:** Regression analysis for variables predicting behavioral intentions and ethical judgment.

Scenario	A			B			C		
	Duplicate submission			Incomplete citation			Failure to cite research		
Peer intention	*β*	*t*		*β*	*t*		*β*	*t*	
Moral equity^a^	0.28	6.05	^***^	0.16	3.13	^**^	0.15	2.57	^***^
Relativism^a^	0.17	3.74	^***^	0.17	3.26	^**^	0.25	4.55	^***^
Egoism^a^	0.09	2.13	^*^	0.13	2.78	^**^	0.05	1.10	
Utilitarianism^a^	0.23	5.54	^***^	0.19	4.34	^***^	0.21	4.65	^***^
Contractualism^a^	0.04	1.10		0.10	2.37	^*^	0.07	1.52	
Overall Model Assessment	*R* = 0.65, *Adj R*^2^ = 0.42, *F* = 88.50^***^			*R* = 0.56, *Adj R*^2^ = 0.31, *F* = 54.63^***^			*R* = 0.58, *Adj R*^2^ = 0.34, *F* = 61.50^***^		
**Self-intention**									
Moral equity^a^	0.26	6.23	^***^	0.30	6.36	^***^	0.20	3.80	^***^
Relativism^a^	0.16	3.85	^***^	0.14	2.90	^*^	0.21	4.12	^***^
Egoism^a^	0.10	2.54	^**^	0.06	1.47		0.01	0.24	
Utilitarianism^a^	0.21	5.63	^***^	0.22	5.37	^***^	0.19	4.69	^*^ ^**^
Contractualism^a^	0.21	5.97	^***^	0.16	4.15	^**^ ^*^	0.24	5.66	^***^
Overall Model Assessment	*R* = 0.73, *R*^2^ = 0.53, *F* = 136.42^***^			*R* = 0.66, *Adj R*^2^ = 0.43, *F* = 92.19^***^			*R* = 0.68, *Adj R*^2^ = 0.45, *F* = 100.37^***^		
**Ethical judgment**									
Moral equity^a^	0.31	7.30	^***^	0.27	5.94	^***^	0.35	7.83	^***^
Relativism^a^	0.11	2.67	^**^	−0.01	−0.26		−0.07	−1.61	
Egoism^a^	−0.03	−0.80		−0.00	−0.05		−0.00	−0.08	
Utilitarianism^a^	−0.02	−0.65		−0.04	−1.06		−0.02	−0.68	
Contractualism^a^	0.26	7.20	^***^	0.38	10.51	^***^	0.32	8.67	^***^
Peer^b^	−0.03	−0.74		−0.01	−0.23		0.04	1.04	
Self^b^	0.28	6.06	^***^	0.25	5.68	^***^	0.29	6.96	^***^
Overall Model Assessment	*R* = 0.76, *Adj R^2^* = 0.57, *F* = 112.38^***^			*R* = 0.71, *Adj R^2^* = 0.50, *F* = 87.06^***^			*R* = 0.78, *Adj R^2^* = 0.61, *F* = 133.65^***^		

a 7 = not consistent with the philosophy to 1 = consistent with the philosophy.

b 7 = would not perform the action to 1 = would perform the action.

^c^7 = unethical to 1 = ethical; d represents Cohen’s d.

* *p* < 0.05;

** *p* < 0.01;

*** *p* < 0.001.

## Discussion

As we expected (H1), students adopted different levels of ethical philosophies when evaluating different AD activities. Overall, the results indicated that students’ moral standards may vary from situation to situation and the finding echoed Yang’s (2012) study. Students adopted strict attitudes toward incomplete citation and failure to cite research published in other countries, as indicated by the fact that all five philosophies in these two scenarios were stronger than in scenario A. In addition, this latitude toward and tolerance of scenario A was also reflected in the students’ behavioral intentions and ethical judgment. Both they and their peers were more likely to engage in duplicate submission, and this act was rated as comparatively ethical.

The inference (H2) that personal traits play important roles in the application of moral philosophy was mostly confirmed. Regarding the differences related to demographic characteristics, first, we found that Taiwanese students had a more pronounced attitude of relativism than Mainland Chinese students across the three scenarios, implying that Taiwanese were strongly affected by their society’s standards. In addition, Taiwanese students possessed a harsh standard with regard to moral equity in scenarios A and B and contractualism in scenarios A and C, revealing that Taiwanese students particularly valued justice and personal obligation. In addition, Mainland Chinese students were more likely to misquote foreign research than Taiwanese students. These differences may be explained by the value system. As we mentioned before, Western values and systems deeply influenced Taiwanese people in many aspects, even in the education system (e.g., Yang, 2019). The Ministry of Education in Taiwan established a well-developed academic integrity rule system at an early stage and committed to promoting the relevant regulations. Therefore, Taiwanese students may have a relatively clear understanding of the scope and definition of academic integrity and are less likely to engage in related behaviors.

Second, females held stricter moral equity and utilitarianism beliefs than males and believed that both they and their peers were less willing to engage in the AD described in each scenario. They also regarded these deviant actions more seriously, consistent with results from previous studies. For example, Pan and Sparks (2012) conducted a meta-analysis of 65 articles and found that females made harsher ethical judgments than males. Interestingly, all students claimed that they were more honest than their peers, and this finding is also consistent with existing research (Engler et al., 2008; Iyer and Eastman, 2008; Yang, 2012; Yang et al., 2013). As mentioned by Yang et al. (2013), students’ misperceptions and awareness of social expectations may have contributed to this finding. Whether females underreport their AD tendencies to conform to social standards needs to be further explored.

Third, the analysis indicated that graduate students relied more on moral equity, relativism, and contractualism in their evaluation of plagiarism behavior in scenarios B and C than undergraduates. They judged these deeds as serious infractions and reported less self-behavioral intention in these activities. A similar strongly condemnatory judgment was reported by Poorolajal et al. (2012). They found that graduate students have more negative attitudes toward plagiarism than undergraduate college students. Thus, it seems that the higher one’s academic level is, the greater value one places on academic ethics. In addition, graduate students took a comparatively strict contractualism-based stance toward misquoting foreign research, further indicating that graduate students value the importance of their obligations even if they are invisible.

One possible explanation of these findings is that college students are relatively unfamiliar with the definition and scope of AD or may have misunderstandings about it. For example, Kwong et al. (2010) found that teachers and students did not reach a consensus regarding the severity of plagiarism and collusion. Without a clear description of AD, instructors can only hold broad discussions of dishonest behavior or list a variety of types of misconduct that might confuse students about the explicit definition of academic integrity (McClung and Schneider, 2015). Such confusion can easily translate into students’ implicit defense of cheating in school, which in turn can provide further justification for cheating (Owunwanne et al., 2010). Empirical evidence reported by Ellahi et al. (2013) demonstrates that the definitional ambiguity of AD is a major factor in academic integrity violations among college students. As the training of graduate students focuses more on research ethics than undergraduate education does, graduate students more accurately and objectively evaluate academic ethical behavior.

Another possible reason why older students are less likely to sanction AD and show less tolerance for ethical deviance than their younger counterparts is maturity (Jurdi et al., 2012). Maturity is also one of the possible reasons that older students are less likely to engage in AD activities (Zhang et al., 2018). This suggestion is in line with Kohlberg’s (1973) theory of moral development, which states that individuals’ moral and ethical reasoning abilities change in predictable ways with age as cognitive abilities develop. With respect to scenario A, which involves only the contributors themselves, the behavior in scenarios B and C significantly impairs the rights of others, revealing the influence of an act having a special relationship with the student’s cognitive perception of right and wrong. In this regard, Ashworth et al. (1997) posited that students’ minimum standard with respect to AD is to not hurt other people. Therefore, considering schools’ relatively broad definition of AD, it is essential for higher education to teach and help students understand the basis of academic integrity.

Finally, with respect to the regression analysis, the results showed that students relied on a combination of philosophies to judge AD behaviors. This result implies that moral philosophy is indeed one of the determinants of behavioral intent and judgment, echoing our third expectation.

For instance, moral equity, relativism, and utilitarianism are good predictors of peers’ AD intentions, and moral equity, relativism, utilitarianism, and contractualism are the common indicators for self-intention in all AD dilemmas. Moreover, moral equity, contractualism, and self-intention are critical indicators of overall ethical judgment across the three scenarios.

The finding that students combine multiple ethical philosophies when deliberating about unethical academic acts echoes Yang’s (2012) study. Yang pointed out that individuals take particular moral philosophies into consideration when assessing the ethics of a situation but also simultaneously use other moral philosophies. Scholars have also found this phenomenon in studies of other moral issues. Lee (2004) evaluated real estate agents’ ethical evaluation, ethical judgment, and behavioral intention and found that their behavioral intention was influenced by different moral philosophies. Lin and Ho (2008) also indicated that students made ethical decisions based on a combination of ethical philosophies, and much evidence has demonstrated that people tend to use mixed moral philosophies when making judgments. On the basis of the extant literature and our findings, it can be found that individuals seem to adopt an integrated ethical approach to dilemmas, including but not limited to AD.

Unexpectedly, peer intention is not the criterion for judging AD conduct. Prior literature has indicated that peer reactions are important for students in assessing academic dishonesty (Ashworth et al., 1997; Mastin et al., 2009), while there is a special relationship between the dishonest behavior of peers and students’ engagement in AD (McCabe et al., 2006, 2008; Taradi et al., 2012). This result indicates that Asian students in higher education may be more independent than we thought, and they may tend to rely on their own perceptions to make judgments.

## Conclusion

Using Reidenbach and Robin’s Multidimensional Ethics Scale, this study designed three scenarios involving dilemmas related to academic dishonesty to examine students’ moral philosophies, behavioral intentions, and ethical judgment. In general, this study provided empirical evidence for employing the MES to examine students’ academic misconduct, and this helped us further realize AD from another perspective. Additionally, we found that the participants’ perceptions of academic ethics differed by geographical area, gender, and education level and that participants integrated multiple moral philosophies to evaluate AD. This implies that according to students’ traits, providing appropriate guidance may be an effective way to enhance students’ academic integrity.

With respect to decreasing students’ AD involvement, establishing a clear definition of AD is a positive way to prevent students from engaging in this type of misconduct, and instructors should also teach students the negative consequences of AD, since the link between students’ perceptions of the severity of AD and the likelihood of engaging in academic misbehavior (e.g., O'Neill and Pfeiffer, 2012; Schuhmann et al., 2013) has been confirmed. In practice, teachers can integrate the concept of academic ethics into each subject instead of offering separate classes. For example, when explaining course requirements, teachers can also explain the relevant knowledge, including the definition, scope, influence, and cases.

In addition, this study made a contribution to understanding Asian students’ moral values and provides a reference for the field, especially regarding which moral philosophy students tend to adopt when encountering ethical dilemmas. It still has some limitations, and the results should be interpreted with caution. First, due to the vast territory of China and the differences among provinces, it is not appropriate to make overgeneralization from the findings to all of China. Additionally, since this is not a large-scale investigation, stating that Taiwanese students have a higher level of academic integrity than Mainland Chinese students is improper. Second, we collected only three types of AD behaviors, which limited our understanding of AD.

Based on these limitations, we suggest that future research adopt the MES to examine more types of AD activities. We hope this study can help those concerned about the issue of academic integrity to have a more complete picture of the current situation because only when we understand the status quo are we able to enhance students’ integrity.

## Data Availability Statement

The original contributions presented in the study are included in the article/supplementary material, further inquiries can be directed to the corresponding authors.

## Ethics Statement

The ethical approval for this study was waived by Taiwan Centers for Disease Control Policy #1010265075 because this study was conducted in a general teaching environment for educational purposes. Due to the consideration of anonymity, we provided informed consent information on the first page of the questionnaire which does not require respondent’s signature, and the respondents started to fill out the questionnaire only after agreeing to participate in the research. No data were collected that could identify specific individuals, and all participants were general adults rather than patients.

## Author Contributions

CH contributed to the conceptualization, formal analysis, methodology, validation, software, writing, and funding acquisition. S-CY contributed to the conceptualization, data curation, project administration, and supervision. C-AY contributed to the writing—review and editing. All authors approved the content of the manuscript and have contributed significantly to the research involved/the writing of the manuscript.

## Funding

This research was financially supported by Humanities and Social Science Youth Foundation of Ministry of Education of the People’s Republic of China (Grant No. 19YJC880034).

## Conflict of Interest

The authors declare that the research was conducted in the absence of any commercial or financial relationships that could be construed as a potential conflict of interest.

## Publisher’s Note

All claims expressed in this article are solely those of the authors and do not necessarily represent those of their affiliated organizations, or those of the publisher, the editors and the reviewers. Any product that may be evaluated in this article, or claim that may be made by its manufacturer, is not guaranteed or endorsed by the publisher.
